# Metabolomic ageing (MileAge) in mid‐life predicts incident vascular, unspecified and all‐cause dementia

**DOI:** 10.1002/alz.71280

**Published:** 2026-05-13

**Authors:** Julian Mutz, Lachlan Gilchrist, Oliver Pain, Petroula Proitsi, Cathryn M. Lewis

**Affiliations:** ^1^ Social, Genetic and Developmental Psychiatry Centre Institute of Psychiatry, Psychology & Neuroscience, King's College London London UK; ^2^ Perron Institute for Neurological and Translational Science Perth Australia; ^3^ Centre for Preventive Neurology Wolfson Institute of Population Health, Queen Mary's University of London London UK; ^4^ Maurice Wohl Clinical Neuroscience Institute Department of Basic and Clinical Neuroscience Institute of Psychiatry, Psychology & Neuroscience, King's College London London UK; ^5^ Department of Medical and Molecular Genetics Faculty of Life Sciences & Medicine King's College London London UK

**Keywords:** ageing, biological age, dementia, metabolomic age, metabolomics

## Abstract

**INTRODUCTION:**

Identifying individuals at risk of dementia is essential for prevention and targeted disease‐modifying strategies. We investigated whether mid‐life metabolomic ageing is associated with incident dementia and its age of onset and assessed joint associations and interactions with *APOE* genotype and dementia polygenic scores.

**METHODS:**

In the UK Biobank, plasma metabolites were quantified at baseline. Metabolomic age (MileAge) delta reflects the difference between metabolite‐predicted and chronological age. Dementia was identified via health records.

**RESULTS:**

Amongst 223,496 participants, 3976 developed dementia. A higher MileAge delta was associated with higher hazards of all‐cause, unspecified and vascular dementia (HR = 1.61, 95% CI 1.28–2.02, *p *= 0.001) and earlier onset. Key metabolites were lipids, lipoproteins and amino acids. MileAge delta and genetic risk were jointly associated with dementia. Individuals with a high MileAge delta and two *APOE* ε4 alleles had a 10.30‐fold higher all‐cause dementia risk (95% CI 7.95‐13.34, *p *< 0.001).

**DISCUSSION:**

Metabolomic ageing and genetic risk likely represent independent biological pathways contributing to dementia risk.

## Highlights

- A metabolite‐predicted age exceeding chronological age (MileAge delta) was associated with significantly higher hazards of vascular, unspecified and all‐cause dementia.
- A higher MileAge delta was also associated with earlier dementia onset, including of Alzheimer's disease.
- Key contributors to the MileAge clock, including lipids, lipoproteins and amino acids, were also associated with dementia risk.
- Individuals with both an older metabolomic age and high genetic risk (e.g., *APOE* ε4/ε4) had markedly elevated dementia risk, suggesting additive effects from metabolic and genetic pathways.

## 1 BACKGROUND

Dementia currently affects an estimated 982,000 individuals in the UK, with projections indicating a rise to 1.4 million by 2040.^1^, ^2^ In 2023, dementia and Alzheimer's disease were the leading causes of death in England and Wales, accounting for 11.6% of all registered deaths.^3^ Although chronological age remains the strongest risk factor, dementia is not an inevitable consequence of ageing. Up to 45% of dementia cases could be delayed or prevented through modifying risk factors.^4^

Effective risk stratification is essential for targeted prevention and disease‐modifying strategies. Despite the long preclinical period of dementia, research has largely focused on individuals later in life, where opportunities to intervene are more limited. Identifying dementia biomarkers in mid‐life could help determine risk earlier, providing greater scope for intervention.

Advances in high‐throughput molecular technologies have made it feasible to quantify hundreds or thousands of biological markers–including metabolites–at scale.^5^ The pathophysiology of most age‐related diseases involves changes in the metabolome.^6^ Metabolomic irregularities include, for example, dysregulated lipid levels and lipoprotein concentrations.^7^ Given that metabolites can be measured in minimally invasive blood samples, metabolomics holds promise for early disease detection.

Emerging evidence suggests that metabolomic profiles can predict dementia risk. For example, in the Whitehall II study, an elastic net model based on serum metabolites modestly improved prediction of dementia across 20 years.^8^ Five metabolites were nominally associated with incident dementia after adjusting for chronological age and sociodemographic factors. Only glucose remained statistically significant after Bonferroni correction, potentially due to its links with neurotoxicity, hyperglycaemia, insulin resistance and vascular injury. In the UK Biobank, metabolomic profiles derived from plasma predicted incident diseases.^9^ The odds of dementia were 6.39‐fold higher in the top 10% of the distribution compared to the bottom 10%. Metabolomic scores developed in the UK Biobank also demonstrated cross‐cohort replicability at predicting four‐year incidence of Alzheimer's disease and vascular/other dementia.^10^

While metabolomic profiles aid risk stratification, biological ageing clocks are conceptually easier to understand, being expressed in unit of years. Ageing clocks involve using machine learning to identify patterns in biological data to predict a person's age^5^ and have been developed using diverse molecular markers, such as DNA methylation, protein abundance or metabolite concentrations. We have previously shown that a metabolite‐predicted age exceeding chronological age was associated with mortality and diverse health and ageing markers.^11^ Ageing clocks also offer certain advantages over clinical predictors such as frailty^12^ because they can be applied to individuals free of diseases, impairments and functional deficits.

To investigate whether metabolomic ageing predicts dementia, we estimated associations between metabolomic age (MileAge) delta and incident Alzheimer's disease, vascular dementia, dementia in other diseases, unspecified dementia and all‐cause dementia, as well as dementia age of onset. To identify specific metabolites that contributed to the MileAge clock and were associated with dementia, we performed metabolome‐wide association analyses. Finally, we examined joint associations and interactions of MileAge delta and genetic risk factors with incident dementia, including apolipoprotein E (*APOE*) genotype and ancestry‐standardized dementia polygenic scores.

## 2 METHODS

### 2.1 Study population

**RESEARCH IN CONTEXT**

1. **Systematic review**: We searched Embase, MEDLINE, PsycINFO, Web of Science and Google Scholar from inception to April 30, 2025 with no language restrictions, using combinations of the following search terms: “metabolomic age”, “biological age”, “metabolomics”, “dementia”, “Alzheimer”, “vascular dementia”, “polygenic score” and “APOE”. Several prospective studies linked individual metabolites or composite metabolomic risk scores to incident dementia. For example, a metabolomic risk score based on eight to nine metabolites predicted Alzheimer's disease, vascular dementia and all‐cause dementia in the UK Biobank, while metabolites modestly improved dementia prediction in the Whitehall II cohort across 21 years of follow‐up. Although metabolomic ageing clocks have been developed and validated against mortality or broad health outcomes, we found no previous study that (i) tested whether a metabolomic ageing clock, defined as the difference between metabolite‐predicted and chronological age, is associated with incident dementia or dementia age at onset, or (ii) evaluated its joint or interactive effects with genetic risk factors such as *APOE* genotype or genome‐wide dementia polygenic scores.
2. **Interpretation**: In 223,496 middle‐aged and older adults from the UK Biobank, we show that a higher metabolite‐predicted age relative to chronological age (MileAge delta) is prospectively associated with higher hazards of vascular, unspecified and all‐cause dementia, and with earlier dementia onset across diagnoses. Key metabolites included lipids, lipoproteins and branched‐chain amino acids. By integrating plasma metabolomics with electronic health‐record and genomic data, we demonstrate that metabolomic ageing and genetic susceptibility (*APOE* genotype and ancestry‐standardized dementia polygenic scores) showed additive but largely independent associations with dementia. To our knowledge, this is the first large‐scale study to evaluate a plasma‐based metabolomic ageing clock against multiple dementia outcomes alongside established genomic risk indicators. Our findings suggest that metabolomic ageing clocks capture distinct biological information relevant to dementia risk.
3. **Future directions**: Future studies should explore the predictive utility of metabolomic ageing clocks relative to, and in combination with, other biological ageing clocks (e.g., epigenetic, proteomic or clinical biochemistry measures) to enhance risk stratification. Longitudinal studies with repeated metabolomic profiling are needed to assess temporal dynamics of MileAge delta and its relationship to neurodegenerative trajectories. Integration with brain imaging, cognitive testing and other biomarkers may clarify whether metabolomic ageing reflects neurobiological changes preceding clinical dementia. Additionally, mechanistic studies could evaluate whether interventions targeting lipid and amino acid metabolism modify metabolomic ageing and, in turn, dementia risk. As plasma‐based clocks are scalable and minimally invasive, their incorporation into mid‐life screening and or application in refining the selection of participants for prevention or disease‐modifying trials warrants investigation.

UK Biobank recruited over 500,000 adults aged 37–73 years,^13^ registered with the UK National Health Service (NHS) and residing within 25‐miles of one of 22 assessment centers. At baseline (2006–2010), participants provided sociodemographic, behavioral and medical information, underwent physical examinations and gave blood and urine samples. Hospital inpatient records are available for most participants; primary care records for ∼230,000. Some participants attended follow‐up assessments. Table S1 lists all data fields used.

### 2.2 Metabolomic ageing (MileAge) clock

Nuclear magnetic resonance (NMR) metabolomic biomarkers were quantified in non‐fasting plasma samples. The Nightingale Health platform measured 168 metabolites in absolute concentration units using a standardized protocol.^14^ Technical variation was removed using the ‘ukbnmr’ R package (algorithm v2).^15^ In a previous study,^11^ we developed a metabolomic clock trained on chronological age using Cubist rule‐based regression.^16^, ^17^ Individual‐level age predictions were aggregated from the outer nested cross‐validation loop test sets to avoid overfitting. Metabolomic age (MileAge) delta was defined as the difference between metabolite‐predicted and chronological age, with positive values indicating an older biological ageing profile. Full details of model training and performance are provided in Mutz, Iniesta and Lewis.^11^

### 2.3 Dementia diagnosis

Incident dementia was defined using ICD‐10 codes for Alzheimer's disease (F00, G30), vascular dementia (F01), dementia in other diseases (F02) and unspecified dementia (F03); all‐cause dementia included any of these. Dementia in other diseases includes dementia in Pick's disease (F020), Creutzfeldt–Jakob disease (F021), Huntington's disease (F022), Parkinson's disease (F023), human immunodeficiency virus disease (F024) and dementia in other specified diseases classified elsewhere (F028). First occurrence dates were ascertained from primary care, hospital inpatient, death registry and self‐reported physician diagnosis data. ICD‐10 codes were extracted from hospital and death records. ICD‐9 codes from hospital records and Read v2 or CTV3 codes from primary care were extracted if they could be mapped to ICD‐10. Administrative, procedure and medication CTV3 codes were not considered. Self‐reported physician diagnosis data were mapped to ICD‐10, with occurrence dates interpolated to mid‐year. Full mapping details for each dementia endpoint are provided in Additional File 1. Dementia subtypes were not treated as mutually exclusive; individuals may receive multiple diagnostic labels, reflecting real‐world diagnostic uncertainty, evolving clinical classification over time and recording patters across routine care data sources. Accordingly, participants could contribute to more than one dementia subtype analysis where multiple diagnostic labels were recorded. The only exception to this approach was that individuals who had a record of unspecified dementia were only included in the analysis of unspecified dementia if they had no other dementia subtype recorded. Follow‐up spanned baseline to dementia diagnosis, loss to follow‐up, death or censoring. Region‐specific censoring dates based on hospital records were October 31, 2022 (England), August 31, 2022 (Scotland) and May 31, 2022 (Wales).

### 2.4 Dementia age of onset

We derived approximate birthdates from birth year and month, assigning random days within each month, accounting for the number of days per month and leap years. Age of onset was calculated as days from birth to first occurrence divided by 365.25.

### 2.5 *APOE* genotype

*APOE* genotype was derived from imputed genotype data using single nucleotide polymorphism (SNPs) rs7412 and rs429358 within the *APOE* gene on chromosome 19, via PLINK v2.0.^18^ SNPs were combined to infer *APOE* alleles (ε1, ε2, ε3 and ε4).^19^ Ambiguous genotypes (ε1ε3 and ε2ε4) were coded as ε2ε4; rare genotypes (ε1ε2 and ε1ε4) were excluded. We defined four risk categories: low (ε2ε2 and ε2ε3), average (ε3ε3), moderate (ε2ε4 and ε4ε3) and high (ε4ε4), with ε3ε3 serving as reference.

### 2.6 Dementia polygenic scores

Genotype quality control steps are reported in Supplementary Methods; genome‐wide association study (GWAS) summary statistics in Table S2. Polygenic scores (PRS) were calculated using GenoPred.^20^ A combined 1000 Genomes Project phase 3^21^ and Human Genome Diversity Project^22^ reference panel, restricted to 1,204,449 HapMap3 variants, was used for linkage disequilibrium estimation. MegaPRS (LDAK v5.1) was used for polygenic scoring, applying the BLD‐LDAK heritability model where SNPs are weighted based on allele frequency, linkage disequilibrium and functional annotations.^23^ MegaPRS models for each dementia outcome were selected using pseudo‐validation. PRS were calculated within assigned ancestry groups (matching individuals to populations in the *N* = 3313 reference data with probability ≥ 0.95) and scaled to units of standard deviation from the matched reference population mean.^24^ PRS subgroups were defined on this scale.

### 2.7 Covariates

A directed acyclic graph (DAG) was used to visualize relationships between the exposure (MileAge delta), outcome (dementia) and select confounders (chronological age, sex, highest educational/professional qualification, cohabitation with spouse/partner, annual gross household income, Townsend deprivation index, fasting time and *APOE* genotype) (Figure S1). Analyses of *APOE* genotype and PRS also included genotype batch number, assessment center and the first six population principal components (derived within UK Biobank) to account for population stratification.^25^

### 2.8 Exclusion criteria

**FIGURE 1:**
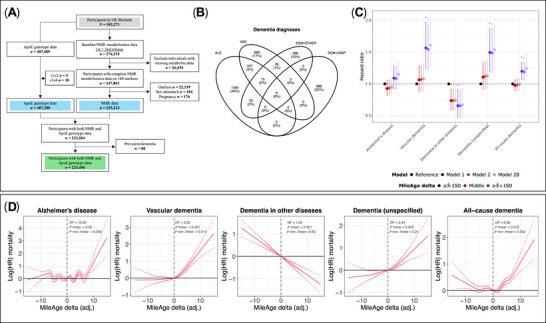
Sample overview and incident dementia. (A) Study sample flowchart. Outliers were defined as metabolite values 4×IQR above or below the median. NMR = nuclear magnetic resonance; IQR = interquartile range. (B) Venn diagram of incident dementia cases. ALZ = Alzheimer's disease; VAD = vascular dementia; DEM OTHER = dementia in other diseases; DEM UNSP = unspecified dementia. (C) Hazard ratios (HR) and 95% confidence intervals from Cox proportional hazards models for incident dementia by MileAge delta. Time since the baseline assessment (in days) was used as the underlying time axis. Reference group: individuals with a MileAge delta smaller than one standard deviation below the mean. Model 1–adjusted for age and sex; Model 2–adjusted for age, sex, highest educational/professional qualification, cohabitation with spouse/partner, annual gross household income, Townsend deprivation index and fasting time; Model 2B–additionally adjusted for apolipoprotein E (*APOE*) genotype. Asterisks indicate statistical significance after correcting *p*‐values for multiple testing using the Benjamini–Hochberg procedure. (D) Log(HRs) and 95% confidence intervals from Cox proportional hazards models for incident dementia, adjusted for Model 2 covariates. Time since the baseline assessment (in days) was used as the underlying time axis. A penalized spline function was applied, with degrees of freedom (DF) selected based on Akaike Information Criterion. Vertical lines indicate the values of the distribution closest to zero (0.00001192219), representing the reference for interpreting the estimates shown. (C and D) *N* = 1881 (Alzheimer's disease); *N* = 933 (vascular dementia); *N* = 512 (dementia in other diseases); *N* = 988 (unspecified dementia); *N* = 3976 (all‐cause dementia).

We excluded women with self‐reported possible pregnancy (i.e., those who responded “yes” or “unsure”) due to altered metabolite profiles, individuals with discordant genetic and self‐reported sex (to mitigate potential data quality issues) and individuals with any missing or outlier metabolite values 4× the interquartile range (IQR) from the median (Figure 1A). We also excluded individuals with dementia onset preceding baseline (i.e., prevalent cases) and those with missing or implausible event dates, including dates prior to, matching or in the same calendar year as their birth date or those that fell outside the follow‐up window. Such exclusions reflect standard quality control procedures to address irregularities in linked health record dates that can arise, for example, from coding errors.

### 2.9 Statistical analyses

Analyses were performed in R (version 4.3.0). Sample characteristics were summarized using means and standard deviations or counts and percentages. We estimated hazard ratios (HRs) and 95% confidence intervals using Cox proportional hazards models to investigate associations between MileAge delta and incident dementia (separate models for Alzheimer's disease, vascular dementia, dementia in other diseases, unspecified dementia and all‐cause dementia). For the primary analysis, MileAge delta subgroups were defined by standard deviation from the mean. Individuals at least one standard deviation below the mean (i.e., with a younger metabolite‐predicted age) served as the reference. Time (in days) since baseline was the underlying time axis. Model 1 was adjusted for chronological age and sex; Model 2 additionally included education, cohabitation, income, deprivation and fasting time; Model 2B was further adjusted for *APOE* genotype. Proportional hazards assumptions were assessed using Schoenfeld residuals. Global and covariate‐specific tests were examined for each dementia outcome. Where there was evidence of modest non‐proportionality, we conducted sensitivity analyses allowing time‐varying effects by including interactions between MileAge delta and centered log‐transformed follow‐up time. These analyses were used to assess robustness rather than to redefine the primary effect estimates. We additionally calculated incidence rates per 1000 person‐years by MileAge delta subgroup for each dementia endpoint to provide absolute incidence context alongside hazard ratios; 95% confidence intervals were derived assuming a Poisson distribution for event counts.

To explore non‐linear relationships between MileAge delta and dementia, we fitted Cox proportional hazard models with penalized splines adjusted for Model 2 covariates. Associations between MileAge delta and dementia age of onset were estimated using ordinary least squares regression, with the covariate adjustment from our primary analysis. To identify metabolites that were important contributors to the MileAge clock and associated with incident dementia, we performed metabolome‐wide association analyses using Cox proportional hazard models (adjusted for Model 2 covariates to reduce the possibility of confounding). We visualized the hazard ratios from these analyses alongside variable importance (VIP) scores previously calculated for MileAge.^11^

We also investigated joint associations and interactions with genetic risk. First, we estimated differences in MileAge delta between *APOE* genotypes and associations with dementia polygenic scores (across and within ancestry groups), using ordinary least squares regression. We then estimated associations between MileAge delta and incident dementia by *APOE* genotype risk category, using Cox proportional hazard models adjusted for age and sex (Model 1) and for all covariates (Model 2). We performed similar analyses for PRS groups, classified into low (≤ 1 SD below the mean), middle and high (≥ 1 SD above the mean) risk categories. Individuals with a MileAge delta in the middle of the distribution in the average *APOE* risk category or middle PRS group served as reference. Given that few incident dementia cases were observed in non‐Europeans (Table S3), these analyses were performed only across ancestry groups. We further estimated pairwise hazard ratio contrasts comparing MileAge delta categories within each *APOE* or polygenic risk group. These contrasts were obtained from the fitted Cox models using marginal means on the hazard ratio scale, with covariates held at their mean values. Finally, to formally test for multiplicative interaction, we included cross‐product terms between MileAge delta (continuous) and *APOE* risk group or PRS (continuous) in the Cox models.

### 2.10 Sensitivity analyses

We performed several analyses to assess the robustness of our findings. We excluded self‐reported physician diagnosis data, as the first occurrence date was approximated and these data are prone to recall error. Individuals with another data source with a matching or subsequent event date were retained. To reduce the possibility of reverse causality, we excluded individuals with incident dementia within two years since baseline and those with less than two years of follow up. We also restricted analyses to those aged 60 years or older at baseline, representing those at higher risk of dementia. Given that only 45% of participants had linked primary care records, we performed a sensitivity analysis within this subset. Dementia may be first or exclusively identified in primary care, potentially resulting in misclassification in the comparison group or inaccurate first occurrence dates. Region and data‐provider specific censoring dates were August 31, 2017 (Wales), May 31, 2017 (England, Vision), March 31, 2017 (Scotland) and May 31, 2016 (England, TPP). To address diagnostic uncertainty, we further excluded individuals with multiple diagnostic labels. This restriction ensured that cases contributed to only a single dementia endpoint, such that, for example, individuals with Alzheimer's diseases were not included in the vascular dementia group. Since certain treatments could impact observed associations, we also excluded individuals using lipid‐modifying treatments at baseline (Table S4).

## 3 RESULTS

### 3.1 Sample characteristics

Among 274,315 participants with metabolomics data, 247,861 had complete data (Figure 1A). Excluding individuals with outlier metabolite values, discordant self‐reported and genetic sex, possible pregnancy, prevalent dementia or missing *APOE* genotype, the sample comprised 223,496 participants (54% female; mean age = 56.97 years, SD = 8.10) (Table 1).

**TABLE 1:** Sample characteristics.

	Analytical sample		
	No dementia (*N *= 219,520)	Incident dementia (*N *= 3976)	Full sample (*N *= 223,496)
MileAge delta, mean (SD)	0.00 (3.77)	−0.06 (3.73)	0.00 (3.77)
Age, mean (SD)	56.82 (8.08)	65.04 (4.32)	56.97 (8.10)
Sex			
Female	118,716 (54.1%)	1885 (47.4%)	120,601 (54.0%)
Male	100,804 (45.9%)	2091 (52.6%)	102,895 (46.0%)
Ethnicity			
White	207,991 (94.7%)	3831 (96.4%)	211,822 (94.8%)
Mixed	1230 (0.6%)	15 (0.4%)	1245 (0.6%)
Black	3103 (1.4%)	45 (1.1%)	3148 (1.4%)
Asian	3773 (1.7%)	43 (1.1%)	3816 (1.7%)
Chinese	631 (0.3%)	2 (0.1%)	633 (0.3%)
Other	1828 (0.8%)	16 (0.4%)	1844 (0.8%)
Missing^a^	964 (0.4%)	24 (0.6%)	988 (0.4%)
Highest qualification			
None	37,034 (16.9%)	1405 (35.3%)	38,439 (17.2%)
O levels/GCSEs/CSEs	59,408 (27.1%)	821 (20.6%)	60,229 (26.9%)
A levels/NVQ/HND/HNC^b^	50,241 (22.9%)	886 (22.3%)	51,127 (22.9%)
Degree	70,364 (32.1%)	777 (19.5%)	71,141 (31.8%)
Missing^a^	2473 (1.1%)	87 (2.2%)	2560 (1.1%)
Household income^c^			
Very low	42,134 (19.2%)	1421 (35.7%)	43,555 (19.5%)
Low	48,375 (22.0%)	919 (23.1%)	49,294 (22.1%)
Medium	49,531 (22.6%)	462 (11.6%)	49,993 (22.4%)
High	38,203 (17.4%)	209 (5.3%)	38,412 (17.2%)
Very high	9682 (4.4%)	42 (1.1%)	9724 (4.4%)
Missing^a^	31,595 (14.4%)	923 (23.2%)	32,518 (14.5%)
Townsend deprivation			
Q1	45,557 (20.8%)	811 (20.4%)	46,368 (20.7%)
Q2	45,405 (20.7%)	782 (19.7%)	46,187 (20.7%)
Q3	44,080 (20.1%)	762 (19.2%)	44,842 (20.1%)
Q4	42,894 (19.5%)	748 (18.8%)	43,642 (19.5%)
Q5	41,315 (18.8%)	869 (21.9%)	42,184 (18.9%)
Missing^a^	269 (0.1%)	4 (0.1%)	273 (0.1%)
Cohabitation			
With partner	160,618 (73.2%)	2752 (69.2%)	163,370 (73.1%)
Single	18,011 (8.2%)	192 (4.8%)	18,203 (8.1%)
Missing^a^	40,891 (18.6%)	1032 (26.0%)	41,923 (18.8%)
Fasting time			
Less than 8 h	210,941 (96.1%)	3833 (96.4%)	214,774 (96.1%)
At least 8 h	8575 (3.9%)	143 (3.6%)	8718 (3.9%)
Missing^a^	4 (0.0%)	0 (0.0%)	4 (0.0%)
Lipid‐modifying treatment			
Yes	175,138 (79.8%)	2247 (56.5%)	177,385 (79.4%)
No	43,386 (19.8%)	1693 (42.6%)	45,079 (20.2%)
Missing^a^	996 (0.5%)	36 (0.9%)	1032 (0.5%)
*APOE* genotype			
ε3ε3	129,685 (59.1%)	1551 (39.0%)	131,236 (58.7%)
ε2ε2	1333 (0.6%)	10 (0.3%)	1343 (0.6%)
ε2ε3	26,920 (12.3%)	265 (6.7%)	27,185 (12.2%)
ε2ε4	5531 (2.5%)	124 (3.1%)	5655 (2.5%)
ε4ε3	51,128 (23.3%)	1591 (40.0%)	52,719 (23.6%)
ε4ε4	4923 (2.2%)	435 (10.9%)	5358 (2.4%)

Abbreviations: CSE, certificate of secondary education; GCSEs, general certificate of secondary education; HNC, higher national certificate; HND, higher national diploma; NVQ, national vocational qualification; SD, standard deviation.

^a^ Missing data may also include “do not know” or “prefer not to answer”.

^b^ Also includes ‘other professional qualifications’.

^c^ Annual household income groups: very low (< £18,000), low (£18,000–£30,999), middle (£31,000–£51,999), high (£52,000–£100,000) and very high (> £100,000).

The median follow‐up of censored individuals was 13.7 years (IQR = 1.4), with up to 2,971,915 person‐years. There were 3976 incident all‐cause dementia cases: 1881 with Alzheimer's disease, 933 with vascular dementia, 512 with dementia in other diseases and 988 with unspecified dementia. Most were identified from hospital records (Table S5); 8.12% (*N* = 323) of participants had more than one recorded dementia diagnosis across data sources or over time, including combinations of Alzheimer's disease, vascular dementia and dementia in other diseases (Figure 1B). Across MileAge delta subgroups, incidence rates for all‐cause dementia ranged from 1.29 to 1.36 per 1000 person‐years. Incidence rates per 1000 person‐years for all dementia outcomes by MileAge delta subgroup are presented in Table S6.

### 3.2 MileAge delta and incident dementia

A metabolite‐predicted age exceeding chronological age was associated with higher hazards of vascular, unspecified and all‐cause dementia (Figure 1C). The age and sex‐adjusted hazard ratio (HR) for all‐cause dementia was 1.20 (95% CI 1.07–1.34, *p* = 0.006). Further adjustment for education, cohabitation status, income, deprivation, fasting time and *APOE* genotype had little impact on these estimates (Table 2). MileAge delta showed the strongest association with vascular dementia (HR = 1.61, 95% CI 1.28–2.02, *p* = 0.001 after full adjustment). No statistically significant associations were identified for Alzheimer's disease (*p*‐values 0.182–0.507). MileAge delta was associated with a lower hazard of dementia in other diseases (HR = 0.67, 95% CI 0.48–0.93, *p* = 0.031). There was evidence of non‐linear associations between MileAge delta and Alzheimer's disease, vascular dementia and all‐cause dementia (Figure 1D). Assessment of proportional hazards assumptions indicated modest evidence of non‐proportionality for MileAge delta in vascular dementia models (global test *p*‐values ranging from 0.015 to 0.046 across models). In analyses allowing time‐varying effects, higher MileAge delta was associated with increased hazards of vascular dementia earlier in follow‐up, with attenuation of this association over time; however, the overall pattern and direction of associations were consistent with the primary analyses.

**TABLE 2:** MileAge delta and incident dementia.

			Model 1				Model 2				Model 2B			
Level	*N*_total_	*N*_incident_	HR	95% CI		*p*	HR	95% CI		*p*	HR	95% CI		*p*
Alzheimer's disease														
≤ $\bar{X}$‐1SD	35656	301	Reference											
Middle	150745	1306	0.93	0.82	1.05	0.366	0.92	0.80	1.07	0.412	0.94	0.81	1.08	0.507
≥ $\bar{X}$+1SD	35000	274	1.09	0.93	1.29	0.412	1.10	0.90	1.33	0.445	1.17	0.96	1.42	0.182
Vascular dementia														
≤ $\bar{X}$‐1SD	35490	135	Reference											
Middle	150075	636	1.06	0.88	1.28	0.574	1.07	0.89	1.29	0.545	1.08	0.90	1.30	0.507
≥ $\bar{X}$+1SD	34888	162	1.57	1.24	1.97	0.001	1.54	1.23	1.94	0.001	1.61	1.28	2.02	0.001
Dementia in other diseases														
≤ $\bar{X}$‐1SD	35464	109	Reference											
Middle	149786	347	0.74	0.59	0.91	0.016	0.74	0.59	0.92	0.016	0.74	0.60	0.92	0.017
≥ $\bar{X}$+1SD	34782	56	0.66	0.48	0.91	0.023	0.65	0.47	0.90	0.023	0.67	0.48	0.93	0.031
Dementia (unspecified)														
≤ $\bar{X}$‐1SD	35490	135	Reference											
Middle	150125	686	1.11	0.92	1.33	0.412	1.12	0.93	1.35	0.366	1.13	0.94	1.36	0.357
≥ $\bar{X}$+1SD	34893	167	1.49	1.19	1.88	0.003	1.49	1.18	1.87	0.003	1.54	1.22	1.93	0.001
All‐cause dementia														
≤ $\bar{X}$‐1SD	35973	618	Reference											
Middle	152192	2753	0.98	0.89	1.06	0.613	0.98	0.90	1.07	0.722	0.99	0.91	1.09	0.894
≥ $\bar{X}$+1SD	35331	605	1.20	1.07	1.34	0.006	1.19	1.06	1.33	0.009	1.24	1.11	1.39	0.001

*Note*: HR, hazard ratio; CI, confidence interval; SD, standard deviation. MileAge delta (years) was categorized as ≤ 1 SD below the mean, middle (± 1 SD), and ≥ 1 SD above the mean. Time since the baseline assessment (in days) was used as the underlying time axis. Model 1–adjusted for age and sex; Model 2–adjusted for age, sex, highest educational/professional qualification, cohabitation with spouse/partner, annual gross household income, Townsend deprivation index and fasting time; Model 2B–additionally adjusted for apolipoprotein E (*APOE*) genotype.

#### 3.2.1 Sensitivity analyses

Excluding self‐reported physician diagnoses (*N* = 8), those with dementia within two years of baseline (*N* = 46) and those with less than two years of follow‐up (*N* = 1089) had negligible impact (Figures S2–S3; Table S7–S8). Estimates were slightly attenuated when restricting to individuals aged ≥ 60 years at baseline (*N* = 96,243; Figure S4; Table S9). In those with linked primary care data (*N* = 101,952; Figure S5), the median follow‐up was 7.43 years (IQR = 1.5). MileAge delta remained nominally associated with vascular dementia (HR = 1.65, 95% CI 1.15–2.37, *p* = 0.077; Figure S6) and with unspecified dementia (Table S10). Excluding individuals with multiple diagnostic labels (*N* = 323) had negligible impact (Figure S7; Table S11). Excluding individuals on lipid‐modifying treatments (*N* = 45,079) modestly attenuated most estimates, although no associations were statistically significant after multiple testing correction (Figure S8; Table S12).

### 3.3 MileAge delta and dementia age of onset

**FIGURE 2:**
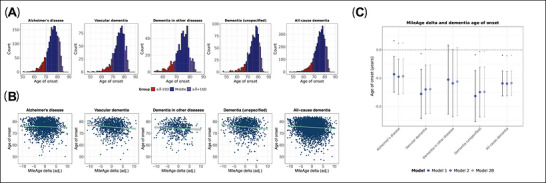
Dementia age of onset. (A) Histograms showing the distribution of dementia age of onset. (B) Scatter plots showing the association between MileAge delta and dementia age of onset. (C) Associations between MileAge delta and dementia age of onset, with betas and 95% confidence intervals estimated using linear regression models. Model 1–adjusted for sex; Model 2–adjusted for sex, highest educational/professional qualification, cohabitation with spouse/partner, annual gross household income, Townsend deprivation index and fasting time; Model 2B–additionally adjusted for apolipoprotein E (*APOE*) genotype. Asterisks indicate statistical significance after correcting *p*‐values for multiple testing using the Benjamini–Hochberg procedure. (A to C) *N* = 1881 (Alzheimer's disease); *N* = 933 (vascular dementia); *N* = 512 (dementia in other diseases); *N* = 988 (unspecified dementia); *N* = 3976 (all‐cause dementia).

Mean age of onset ranged from 74.24 years (SD = 5.20) for dementia in other diseases to 75.57 years (SD = 5.06) for Alzheimer's disease (Figure 2A; Table S13). Higher MileAge delta was associated with an earlier onset of Alzheimer's disease (*β =* –0.09, 95% CI ‐0.15 to ‐0.03, *p* = 0.004), vascular dementia (*β* = –0.14, 95% CI ‐0.22 to ‐0.05, *p* = 0.003), unspecified dementia (*β* = –0.15, 95% CI ‐0.24 to ‐0.06, *p* = 0.002) and all‐cause dementia (*β* = –0.12, 95% CI ‐0.16 to ‐0.07, *p* < 0.001) (Figures 2B and C). Covariate adjustment did not materially impact association estimates (Table 3).

**TABLE 3:** MileAge delta and dementia age of onset.

		Model 1				Model 2				Model 2B			
	*N*	*β*	95% CI		*p*	*β*	95% CI		*p*	*β*	95% CI		*p*
Alzheimer's disease	1881	−0.09	−0.15	−0.02	0.009	−0.09	−0.16	−0.03	0.004	−0.09	−0.15	−0.03	0.004
Vascular dementia	933	−0.16	−0.24	−0.07	0.001	−0.14	−0.23	−0.05	0.003	−0.14	−0.22	−0.05	0.003
Dementia in other diseases	512	−0.11	−0.23	0.02	0.090	−0.12	−0.24	0.00	0.060	−0.11	−0.23	0.01	0.072
Dementia (unspecified)	988	−0.16	−0.25	−0.07	0.001	−0.15	−0.24	−0.06	0.002	−0.15	−0.24	−0.06	0.002
All‐cause dementia	3976	−0.12	−0.16	−0.07	<0.001	−0.12	−0.16	−0.08	<0.001	−0.12	−0.16	−0.07	<0.001

*Note*: CI, confidence interval. Model 1–adjusted for sex; Model 2–adjusted for age, sex, highest educational/professional qualification, cohabitation with spouse/partner, annual gross household income, Townsend deprivation index and fasting time; Model 2B–additionally adjusted for apolipoprotein E (*APOE*) genotype.

### 3.4 Metabolome‐wide association analyses

**FIGURE 3:**
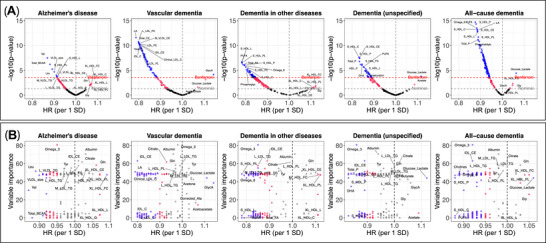
Metabolome‐wide associations. (A) Volcano plots showing associations between metabolite levels and incident dementia. Metabolites were standardized to have a mean equal to zero and a standard deviation (SD) of one. Hazard ratios (HR) were estimated using Cox proportional hazards models, with days since the baseline assessment as the underlying time axis. Analyses were adjusted for age, sex, highest educational/professional qualification, cohabitation with spouse/partner, annual gross household income, Townsend deprivation index and fasting time. *P*‐values were ‐log10 transformed. (B) Scatter plots showing HRs for incident dementia associated with a one standard deviation difference in metabolite levels and MileAge variable importance (VIP) scores. VIPs represent averages across the 10 outer loops of the nested cross‐validation and are the same across the five panels. Metabolites with statistically significant associations with incident dementia (based on their HRs) are shown in red and purple, indicating nominal and Bonferroni‐corrected significant levels, respectively. (A and B) *N* = 223,496.

Amongst 69 metabolites nominally associated with Alzheimer's disease (*p* < 0.05), eight remained statistically significant after Bonferroni correction: leucine, valine, total branched‐chain amino acids and certain lipoprotein measures—all associated with a lower hazard of Alzheimer's disease (Figure 3A). Of the 101 metabolites nominally associated with vascular dementia, 72 survived multiple testing correction. GlycA was the only metabolite associated with a higher hazard of vascular dementia (HR = 1.14, 95% CI 1.07‐1.22, *p* < 0.001), while lipids and lipoproteins were associated with a lower hazard (estimates ranging from HR = 0.79 for linoleic acid to HR = 0.88 for very low‐density lipoprotein phospholipids). None of the 54 metabolites associated with dementia in other diseases after Bonferroni correction had a HR > 1; lipids and lipoproteins were associated with a HR < 1 (e.g., polyunsaturated fatty acids: HR = 0.78, 95% CI 0.71‐0.86, *p* < 0.001). After multiple testing correction, 43 and 106 metabolites remained statistically significantly for unspecified and all‐cause dementia. Glucose‐lactate, a composite measure that accounts for anaerobic glycolysis between sample collection and processing, was associated with higher hazards of unspecified (HR = 1.12, 95% CI 1.05‐1.18, *p* < 0.001) and all‐cause dementia (HR = 1.06, 95% CI 1.03‐1.09, *p* < 0.001). Lipids, lipoproteins and amino acids (leucine, valine and total branched‐chain amino acids) were associated with lower hazards of all‐cause dementia (Additional File 2).

Certain metabolites associated with dementia also strongly impacted the predictive accuracy of MileAge (Figure 3B). Omega 3 had the highest VIP score of 80.8 and was inversely associated with Alzheimer's disease, dementia in other diseases, unspecified dementia and all‐cause dementia (at *p* < 0.05/168). Albumin had the second highest VIP score (70.6) but was not associated with dementia after multiple testing correction. Intermediate‐density lipoprotein cholesteryl esters (VIP = 63.0) were associated with lower hazards of vascular, unspecified and all‐cause dementia (Bonferroni‐corrected). However, there was no strong correlation between VIP scores and dementia hazard ratios. For example, polyunsaturated fatty acids were associated with all dementia outcomes but had a VIP score of zero (Additional File 2).

### 3.5 MileAge delta and dementia polygenic scores

**FIGURE 4:**
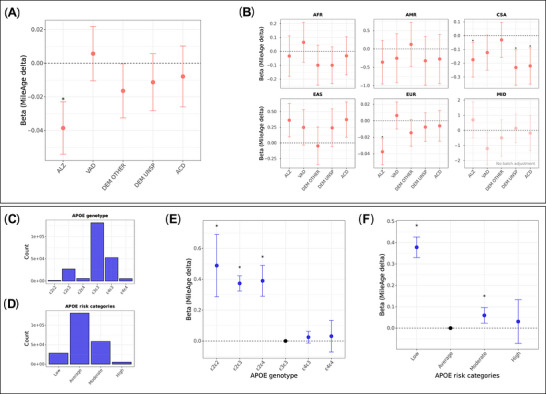
Dementia polygenic scores and *APOE* genotype. (A) Associations between dementia polygenic scores and MileAge delta. ALZ = Alzheimer's disease; VAD = vascular dementia; DEM OTHER = dementia in other diseases; DEM UNSP = unspecified dementia; ACD = all‐cause dementia. *N* = 219,481. (B) Associations between dementia polygenic scores and MileAge delta by ancestry population. AFR = African; AMR = Admixed American; EAS = East Asian; EUR = European; CSA = Central and South Asian; MID = Middle Eastern. *N* = 3426 (AFR); *N* = 278 (AMR); *N* = 3916 (CSA); *N* = 1012 (EAS); *N* = 210,755 (EUR); *N* = 94 (MID). (C) Bar plot showing the distribution of apolipoprotein E (*APOE*) genotypes. (D) Bar plot showing the distribution of *APOE* genotypes classified into low (ε2ε2 and ε2ε3), average (ε3ε3), moderate (ε2ε4 and ε4ε3) and high (ε4ε4) risk categories. (E) Associations between *APOE* genotypes and MileAge delta. (F) Associations between *APOE* genotype risk categories and MileAge delta. (A, B, E and F) Estimates shown are ordinary least squares regression beta coefficients and 95% confidence intervals. Models were adjusted for the first six population principal components, genotype batch number, assessment center, age, sex, highest educational/professional qualification, cohabitation with spouse/partner, annual gross household income, Townsend deprivation index and fasting time. Asterisks indicate statistical significance after correcting *p*‐values for multiple testing using the Benjamini–Hochberg procedure. (C to F) *N* = 223,496.

Amongst 219,481 individuals with genotype data, polygenic scores (PRS) for Alzheimer's disease were associated with a metabolite‐predicted age younger than chronological age (*β* = –0.039, 95% CI ‐0.054 to ‐0.023, *p* < 0.001) (Figure 4A). The magnitude of this association corresponded to a difference in MileAge delta of about two weeks per one standard deviation difference in PRS (Table S14). In ancestry‐specific analyses, PRS for Alzheimer's disease were associated with a younger metabolite‐predicted age in Europeans (*β* = –0.038, 95% CI ‐0.053 to ‐0.022, *p* < 0.001) and Central/South Asians (*β* = –0.174, 95% CI ‐0.300 to ‐0.047, *p* = 0.019) and with a nominally older metabolite‐predicted age in East Asians (*β* = 0.363, 95% CI 0.096‐0.630, *p* = 0.052) (Figure 4B). The non‐European estimates corresponded to differences in MileAge delta of about two and four months, respectively, per standard deviation difference in PRS. In Central/South Asians, PRS for unspecified and all‐cause dementia were associated with a younger metabolite‐predicted age (*β* = –0.230, 95% CI ‐0.359 to ‐0.102, *p* = 0.005 and *β* = –0.219, 95% CI ‐0.350 to ‐0.087, *p* = 0.005) (Table S15).

### 3.6 MileAge delta and *APOE* genotype

*APOE* genotype and risk category distributions are shown in Figures 4C and D. *APOE* genotypes ε2ε2, ε2ε3 and ε2ε4 were associated with a metabolite‐predicted age older than chronological age, with betas ranging from 0.373 to 0.489 (Figure 4E; Table S16). Compared to average risk (ε3ε3), low and moderate risk, but not high risk, was associated with a MileAge exceeding chronological age (Figure 4F).

### 3.7 MileAge delta and incident dementia by *APOE* genotype

**FIGURE 5:**
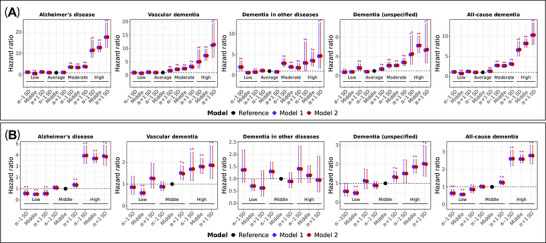
MileAge delta and incident dementia by *APOE* genotype and dementia PRS. (A) Hazard ratios and 95% confidence intervals from Cox proportional hazards models for incident dementia by MileAge delta and apolipoprotein E (*APOE*) genotypes. *APOE* genotypes were classified into low (ε2ε2 and ε2ε3), average (ε3ε3), moderate (ε2ε4 and ε4ε3) and high (ε4ε4) risk categories. Time since the baseline assessment (in days) was used as the underlying time axis. Reference group: individuals in the average *APOE* risk category with a MileAge delta in the middle of the distribution. Model 1–adjusted for the first six population principal components, age and sex; Model 2–adjusted for the first six population principal components, age, sex, highest educational/professional qualification, cohabitation with spouse/partner and annual gross household income. No adjustment was made for genotype batch number, assessment center, Townsend deprivation index and fasting time due to sparse data. SD = standard deviation. (B) Hazard ratios and 95% confidence intervals from Cox proportional hazards models for incident dementia by MileAge delta and dementia polygenic scores (PRS). PRS were classified into low (≤ 1 SD below the mean), middle and high (≥ 1 SD above the mean) risk categories. Time since the baseline assessment (in days) was used as the underlying time axis. Reference group: individuals with a PRS and MileAge delta in the middle of the distribution. Model 1–adjusted for the first six population principal components, genotype batch number, assessment center, age and sex; Model 2–adjusted for the first six population principal components, genotype batch number, assessment center, age, sex, highest educational/professional qualification, cohabitation with spouse/partner, annual gross household income, Townsend deprivation index and fasting time. (A and B) Asterisks indicate statistical significance after correcting *p*‐values for multiple testing using the Benjamini–Hochberg procedure. Sample sizes reported in Tables S18 and S20.

Individuals with ε2ε2/ε2ε3 genotypes had lower hazards of dementia (HRs between 0.70 and 0.96), whereas those with at least one ε4 allele had higher hazards of dementia (up to HR = 12.53, 95% CI 11.37‐13.81, *p* < 0.001 for Alzheimer's disease) (Figure S9; Table S17). Individuals in the moderate (ε2ε4 and ε4ε3) and high (ε4ε4) *APOE* risk groups had elevated hazards of dementia (Table S18). Hazards were generally highest in those with a metabolite‐predicted age exceeding chronological age and lowest in those with a younger metabolite‐predicted age (Figure 5A). For example, the hazard ratios for all‐cause dementia were 3.02 (95% CI 2.63‐3.48, *p* < 0.001) and 10.30 (95% CI 7.95‐13.34, *p* < 0.001) for individuals with a metabolite‐predicted age that exceeded chronological age in the moderate and high *APOE* risk groups, respectively. In pairwise contrast analyses derived from the same cross‐classified models, differences between MileAge delta categories within *APOE* risk groups were most apparent for the low and average *APOE* risk groups (Table S19).

### 3.8 MileAge delta and incident dementia by dementia polygenic scores

About 80% of hazard ratios were below one for individuals with low PRS; 58% of these estimates were statistically significant (Table S20). In the middle PRS group, individuals with a MileAge exceeding chronological age had higher hazards of Alzheimer's disease, vascular, unspecified and all‐cause dementia. Individuals in the high PRS group generally had higher hazards of dementia (except for dementia in other diseases) across MileAge delta subgroups. The strongest associations were observed for Alzheimer's disease in those with a high PRS across MileAge delta groups (HRs from 3.70 to 3.97) (Figure 5B). Pairwise contrasts comparing MileAge delta categories within each PRS stratum are presented in Table S21.

### 3.9 Cross‐product interactions

We identified only one nominally significant interaction between MileAge delta and *APOE* genotype, suggesting that a one‐year higher MileAge delta was associated with 17% higher hazards of dementia in other diseases in the high vs low *APOE* risk groups (Table S22). No statistically significant interactions with dementia PRS were observed (Table S23).

## 4 DISCUSSION

In over 220,000 individuals, a metabolite‐predicted age (MileAge) exceeding chronological age was associated with higher hazards of vascular, unspecified and all‐cause dementia. Individuals with a MileAge delta greater than one standard deviation above the mean had 20% higher hazards of all‐cause dementia than those with a MileAge delta at least one standard deviation below the mean. A higher MileAge delta was also associated with an earlier dementia onset, including of Alzheimer's disease. Lipids, lipoproteins and amino acids importantly contributed to the MileAge clock and were associated with dementia. Genetic risk factors, including *APOE* genotype and dementia polygenic scores, and MileAge delta were jointly associated with dementia, with little evidence of multiplicative interactions. Individuals with a metabolite‐predicted age exceeding their chronological age and a dementia polygenic score greater than one standard deviation above the mean or two copies of the *APOE* ε4 allele had 1.9 to 11‐fold higher hazards of vascular dementia.

Our findings broadly align with prior studies linking metabolites^26^ and metabolomic profiles^9^ to dementia. Our study highlights the role of metabolomic ageing in dementia, especially vascular dementia. The lack of statistically significant association between MileAge delta and Alzheimer's disease contrasts with studies that identified metabolites linked to Alzheimer's disease risk.^26^ Given the role of dysregulated lipid metabolism in Alzheimer's disease,^27^ this finding is unexpected. Notably, we identified fewer metabolites associated with Alzheimer's disease than with other dementia outcomes. PhenoAge, a second‐generation ageing clock trained on physiological dysregulation, was associated with Alzheimer's disease (HR = 1.04 per 1‐year increase in PhenoAge) in a prior study of 30 incident cases.^28^ Our metabolome‐wide analysis identified branched‐chain amino acids as inversely associated with Alzheimer's disease, which is consistent with prior research.^26^

An inverse association was observed between MileAge delta and incident dementia in other diseases. This finding should be interpreted with caution, as this outcome represents a heterogeneous group of secondary dementias rather than primary neurodegenerative disorders. In our data, this category was dominated by dementia in Parkinson's disease (F02.3) and dementia in other specified diseases (F02.8), with relatively few cases of dementia in Pick's or Huntington's disease. These conditions differ in their underlying biology, clinical course and timing of diagnosis, and this pattern may not necessarily indicate a protective effect of higher metabolomic age.

The association between MileAge delta and earlier dementia onset highlights the potential of the MileAge clock to predict not only dementia risk but also timing of disease onset. This is relevant because of the long preclinical period of neurodegenerative diseases. Notably, MileAge delta was associated with an earlier Alzheimer's disease onset, despite not being associated with incident Alzheimer's disease. This could suggest that the MileAge clock captures indicators of speed of progression of Alzheimer's disease. While this finding warrants cautious interpretation and replication in independent cohorts, it broadly aligns with findings from deCODE genetics showing that proteomic risk scores capture short‐term disease processes, while genetic risk scores capture long‐term disease risk.^29^

Our study also provides insights into the joint association of genetic risk and metabolomic ageing with incident dementia. We did not find that a higher genetic risk (assessed through *APOE* genotype and dementia polygenic scores) was associated with a metabolite‐predicted age exceeding chronological age. Instead, both dementia polygenic scores and *APOE* genotype were modestly associated with a MileAge younger than chronological age. Interpretation of this finding should be cautious, as selection into UK Biobank is known to be influenced by survival and overall health, and such selection processes may induce collider bias if genetic risk and metabolomic ageing jointly affect participation or survival to recruitment. Given that genetic risk and MileAge delta were independently associated with dementia, our results indicate that metabolomic ageing does not mediate the relationship between genetic risk and dementia. Rather, genetic risk and MileAge delta appear to represent separate, additive pathways associated with dementia risk.

Our study has certain limitations. Dementia diagnoses were ascertained from routine care and likely include some misclassification. Moreover, ascertainment relied on multiple data sources (hospital inpatient, death registry, primary care and self‐reported physician diagnosis), which may vary by healthcare utilization, age at diagnosis and disease severity. For example, dementia recorded in hospital inpatient or death registry data may be more likely to reflect later‐stage disease, greater comorbidity burden or more severe presentations, whereas dementia recorded exclusively in primary care may represent earlier or less severe disease. We addressed this through sensitivity analyses excluding self‐reported physician diagnosis data and individuals with multiple diagnostic labels—a common occurrence in dementia.^30^ Some of these concerns could be addressed through changes in diagnostic practice towards biological criteria^31^; but see.^32^ To reduce potential under‐ascertainment of dementia cases captured primarily in primary care, we performed sensitivity analyses restricted to participants with linked primary care records, although follow‐up was shorter in this subset. However, linkage to primary care data was available only for a subset of participants and is unlikely to be missing at random. Competing risks were considered in a sensitivity analysis excluding those with multiple diagnostic labels; due to diagnostic uncertainty in dementia,^33^ no competing risk models were fitted. Although effect estimates were attenuated in some restricted samples, the overall pattern of associations was broadly consistent across sensitivity analyses, suggesting that our findings are unlikely to be explained solely by differential diagnostic capture, while acknowledging that residual ascertainment bias cannot be fully excluded when using routinely collected health data. Our analyses of dementia age of onset are subject to potential collider bias, as they are necessarily restricted to individuals with an observed dementia diagnosis during follow‐up. Conditioning on dementia diagnosis may distort associations between metabolomic ageing and age at diagnosis if metabolomic ageing and other factors that influence dementia incidence, survival or diagnostic capture jointly affect the probability of being observed as a dementia case. For example, metabolomic ageing is associated with mortality^11^ and potentially competing health conditions, which may influence whether and when dementia is diagnosed, thereby shaping the distribution of age at diagnosis among observed cases. Consequently, associations with dementia age of onset should be interpreted cautiously as descriptive rather than causal. The coverage of the Nightingale Health platform used to develop the MileAge clock is lipid and lipoprotein focused, omitting certain metabolites linked to ageing. Most participants only had metabolomics data for a single time point. Longitudinal studies with repeated measures could clarify the temporal relationship between metabolomic ageing and dementia onset. Both the development of the metabolomic ageing clock and the analyses of dementia risk were performed within UK Biobank, such that any cohort‐specific biases may influence both steps and limit the external validity of our findings; replication in independent cohorts will be important to assess generalizability. Our study's observational nature precludes causal inference; reverse causality cannot be ruled out despite our efforts to address it through a sensitivity analysis excluding the first two years of follow‐up. While we adjusted for key confounders, residual confounding may persist. Future studies could provide a comparison between MileAge delta and other ageing markers, investigating whether integration with other markers improves dementia risk prediction.

## 5 CONCLUSION

A metabolite‐predicted age that exceeds chronological age was associated with higher hazards of vascular, unspecified and all‐cause dementia, as well as earlier dementia onset, including of Alzheimer's disease. These findings suggest that MileAge delta could help identify individuals at risk before clinical symptoms emerge. Future risk stratification incorporating both genetic and modifiable factors, such as metabolomic age, may enable more targeted preventative strategies.

## AUTHOR CONTRIBUTIONS

Julian Mutz conceived the idea of the study, acquired the data, carried out the analysis, interpreted the findings and wrote the manuscript. Lachlan Gilchrist and Oliver Pain provided methodological support. Lachlan Gilchrist, Oliver Pain, Petroula Proitsi and Cathryn M Lewis reviewed and edited the manuscript. All authors read and approved the final manuscript.

## CONFLICT OF INTEREST STATEMENT

Cathryn M Lewis is a member of the scientific advisory board of Myriad Neuroscience, has received speaker fees from SYNLAB and received consultancy fees from UCB Pharma. Oliver Pain provides consultancy services for UCB Pharma. All other authors declare no conflict of interest. Author disclosures are available in the supporting information.

## ETHICAL APPROVAL

Ethical approval for the UK Biobank study has been granted by the National Information Governance Board for Health and Social Care and the NHS North West Multicentre Research Ethics Committee (11/NW/0382). No project‐specific ethical approval is needed.

## CONSENT STATEMENT

All human subjects provided informed consent.

## Supporting information



Supporting Information

Supporting Information

Supporting Information

Supporting Information

## Data Availability

The data used are available to all bona fide researchers for health‐related research that is in the public interest, subject to an application process and approval criteria. Study materials are publicly available online at http://www.ukbiobank.ac.uk.
